# A Rapid Scoping Review of Resilience Facilitators and Barriers among Black Children and Young People in Canada

**DOI:** 10.1177/08445621261451565

**Published:** 2026-05-14

**Authors:** Emmanuel A. Marfo, Albert Junior Frimpong, Haris Musavar, Bukola Salami

**Affiliations:** 1 Department of Community Health Sciences, Cumming School of Medicine, 70401 University of Calgary, Calgary, Alberta, Canada; 2 3158 Faculty of Nursing, University of Alberta, Edmonton, Alberta, Canada

**Keywords:** resilience, Black children and youth, anti-Black racism, strengths-based practice, well-being, Canada

## Abstract

**Background and Purpose:**

Despite pervasive structural and systemic adversities, Black children and youth in Canada demonstrate significant resilience. However, mainstream discourse often privileges deficit-based narratives, marginalizing these strengths. This rapid scoping review identifies and synthesizes the breadth and depth of available evidence on resilience among Black young people in Canada to counter these stereotypes and inform culturally responsive interventions.

**Methods:**

This rapid scoping review utilized four electronic databases, ProQuest (gray literature), and a supplemental Google Scholar search. Two independent reviewers performed screening and data extraction. Findings were synthesized using descriptive statistics and narrative description.

**Results:**

A total of 24 reports, including 23 peer-reviewed articles, and one thesis met the inclusion criteria. Our findings reveal: (a) geographic (Ontario-centric) and methodological imbalances; (b) that most studies contextualized resilience around intersecting structural and systemic adversities, including anti-Black racism in schools, socioeconomic hardship, and mental health barriers; (c) that research primarily explored resilience capacities and processes, with less attention given to outcomes; (d) that resilience was supported by a dynamic combination of individual, relational, and community-level factors; and (e) that barriers to resilience spanned systemic, interpersonal, and individual levels, with racial discrimination and structural racism acting as pervasive obstacles.

**Conclusions:**

Resilience among Black youth is a dynamic process of resistance to structural racism, not just a personal trait. While matrifocal networks and spirituality are powerful facilitators, they are frequently hindered by systemic exclusion in education and healthcare. Future efforts must prioritize structural reforms and relational nursing care.

## Background and Purpose

Canada is a diverse ethnocultural country offering a wide range of socioeconomic opportunities. Yet, Black children and youth, including those from African, Caribbean, and other Black communities, navigate these opportunities amid systemic and structural challenges (Alumona et al., 2025; Fante-Coleman & Jackson-Best, 2020). These include anti-Black racism within education and health systems and socioeconomic exclusion, which disproportionately affect Black young people and shape their sense of belonging, identity, and aspirations (Alumona et al., 2025; Cénat et al., 2025; Salami et al., 2022; Williams et al., 2024). Despite these inequities, Black children and youth consistently demonstrate significant strengths and resilience (Grant et al., 2023). These assets, however, are frequently overlooked in mainstream discourse, which continues to privilege deficit-based narratives and negative stereotypes (Jones et al., 2023; Quince, 2022).

Resilience is understood as an evolving process that encompasses a constellation of competencies and outcomes that enable individuals to adapt, cope, and succeed despite sustained systemic adversity (Marfo et al., 2025). The need for evidence mapping on Black children and youth resilience in Canada and the United States has been previously documented and is currently being addressed through a larger cross-border Canada and United States scoping review (Marfo et al., 2025). Importantly, no synthesized evidence currently examines strategies, facilitators, and barriers shaping resilience within this population in Canada. Existing reviews have largely examined Black youth in general terms without centering resilience or have focused on mental health outcomes without mapping the mechanisms through which resilience is enacted (Alumona et al., 2025; Martínez-Vega et al., 2024; Olanlesi-Aliu et al., 2023). This gap may, in part, reflect an emphasis on risk and pathology, which has historically overshadowed strengths-based and culturally grounded constructs. As a result, understanding of resilience among this population remains limited, and the development of culturally responsive interventions grounded in the lived realities of Black communities is constrained.

Although the large cross-border review is underway (Marfo et al., 2025), a critical and immediate gap was identified in the Canadian context. Studies of Black young people in Canada often extend beyond the strict Statistics Canada definition of youth, which includes ages 15 to 24 (Statistics Canada, 2024). Consequently, relevant evidence on young Black adults falls outside the inclusion criteria of the planned review. To avoid overlooking this Canada-context-specific literature, a rapid synthesis was warranted.

Therefore, the purpose of this rapid scoping review was to identify, synthesize, and describe the breadth and depth of available evidence on resilience among Black young people in Canada. This review contributes to nursing, health, and interdisciplinary scholarship by identifying key structural and social factors across individual, relational, community, and cultural levels that foster or constrain resilience. By elucidating these factors, the review offers practice-relevant insights to support nurses’ roles in assessment, health promotion, advocacy, and the design of culturally responsive, trauma- and equity-informed interventions aimed at enhancing the well-being of Black children and youth in Canadian healthcare and community settings.

## Methods and Procedures

### Design

This scoping review was guided by the methodological framework for scoping reviews (Arksey & O’Malley, 2005), as enhanced by Levac and collegaues (Levac et al., 2010), and followed the PRISMA Extension for Scoping Reviews (Tricco et al., 2018). This includes five steps namely: (1) formulating a research question; (2) identifying relevant studies; (3) selecting studies; (4) organizing and extracting data; and (5) compiling, summarizing, and reporting the findings. Given the urgent need for Canada-context specific data, our approach was modified to be a rapid scoping review.

This approach was considered appropriate because the larger cross-border project is extensive and will require significant amount of time to complete. Importantly, the rapid review on this topic was necessary due to recent and evolving policy changes in equity, diversity, and inclusion (EDI) that may affect the resilience and well-being of Black children and youth in Canada. For example, Bill 13 in Alberta restricts mandatory EDI, anti-racism, and cultural competency training for regulated professionals (Government of Alberta, 2025). Olatubi et al. (2026) situated Alberta's Bill 13 within broader national policy shifts affecting EDI in health care, arguing that such developments raise concerns about the future of equity-oriented and culturally safe practice. Similarly, cross-border policy shifts, including reductions in EDI-related funding during President Trump's second administration (The White House, 2025), further underscore the need for timely evidence to inform policy and practice. The rapid scoping review design enabled us to capture the current policy context, identify key facilitators and barriers to resilience, and generate actionable insights, while explicitly acknowledging that findings are descriptive and indicative rather than exhaustive. Despite the rapid approach, methodological rigor was preserved through team-based screening, consensus-based data extraction, and adherence to PRISMA-ScR reporting standards.

Consequently, the sixth step recommended by Levac (Levac et al., 2010), which includes stakeholder consultation, was omitted due to the rapid nature of this review. The omission of this stage situates the study's findings to published and grey literature, without contextual insights from Black children, youth, families, or community stakeholders.

In addition, our approach to this review was informed by the intersectionality theoretical lens (Crenshaw, 1989), which assists in understanding how overlapping systems of oppression, such as race, class, gender, immigration status, and age, interact to shape the lived experiences of Black children and youth, including their resilience. Our research team is diverse and includes Black and racialized members with nursing backgrounds, migration ties to Ghana, Nigeria, and Afghanistan, and representation across multiple generations, including young people. These positionalities strengthen the inquiry by enabling culturally grounded interpretation, contextualized understandings of resilience, and reflexive analysis that centers the lived realities and strengths of Black children and young people.

The protocol for the larger scoping review (Marfo et al., 2025) was adapted for this rapid review. The modified protocol for the rapid review was not registered. The following sections detail the execution of these five steps.

### Research Question

We sought to answer the question: What individual, community, relational, and cultural factors facilitate or hinder resilience among Black children and young people in the context of systemic adversity in Canada?

### Identification of Relevant Studies: Search Strategy

On July 16, 2025, studies on resilience among Black children and young people were identified by searching four comprehensive electronic databases including CINAHL and Ovid MEDLINE, which focused on health (i.e., medicine, clinical sciences, public health and epidemiology, nursing, biomedical sciences, health services research, psychology and behavioral sciences, and medical education) and ERIC, and PsycINFO, which focus on education and psychology (e.g., educational psychology, gender, sexuality, cultural studies, stress and resilience, social work, and developmental psychology). We also searched ProQuest Dissertations and Theses to identify relevant gray literature. The comprehensive search terms were developed and applied with the help of a research librarian (see (Marfo et al., 2025) for comprehensive search strategy).

We used the Population, Concept, Context criteria (PCC) (Chan et al., 2024) to determine eligible studies. The Population (P) was defined as studies focused on Black children and young people (including African, African-Canadian, and Caribbean descendants). While the initial protocol search adhered to a developmental age range of 0–24, this rapid scoping review adopted a more inclusive approach. *Children* were defined as individuals under 18 years of age. *Youth* and *young people* were defined using a context-sensitive approach consistent with equity-oriented research, where these terms may extend beyond chronological age. Studies were included if authors explicitly identified their study population as children, youth, or young people, being attentive to prolonged and non-linear transitions to adulthood shaped by structural inequities, transnational and cultural influences, migration, and socio-economic conditions. To ensure the review captured the full breadth of the Canadian experience, eligibility was not constrained by a rigid upper age limit, even where samples extended into young adulthood (Arnett, 2000). Studies were eligible if they focused exclusively on this population without any specific age limit. The Concept (C) was resilience, which included process, capacities, and outcomes. We defined resilience processes as a dynamic process of adapting to, coping with, or resisting adversity over time, resilience capacities as individual, relational, or contextual characteristics that support or promote positive adaptation in the face of adversity, and resilience outcomes as observable behavioral, psychological, developmental, or social manifestations of resilience (Marfo et al., 2025). The Context (C) mandated that studies must be conducted in, or explicitly address, the Canadian context. Additionally, a supplemental Google Scholar search was conducted after modifying the population criteria to extend the age range on September 30^th^, 2025. The Google Scholar search was used to identify recently published and in-press studies aligned with the revised age range.

Only primary original research, including quantitative, qualitative, or mixed method reports, published in English from 2000 to present was included in this review. We excluded French and non-English reports. As a rapid scoping review, prioritizing English-language reports enabled timely synthesis of the evidence to inform research and policy in the context of urgent changes to EDI and equity policies affecting Black children and youth. While this decision reflects practical resource constraints, we acknowledge the transnational nature of Black communities and Canada's bilingual context. Consequently, valuable perspectives, particularly from Francophone Canadians, may be missed. We also excluded non-research reports (e.g., editorials, commentaries, and opinions) and conference abstracts.

### Selection of Studies

Two authors (H. M. and A.-J. F.) independently conducted Level 1 (title and abstract) and Level 2 (full-text) screening using predefined inclusion criteria in Covidence systematic review software. During Level 2 full-text screening, reviewers documented reasons for exclusion for each report based on failure to meet the Population, Concept, or Context (PCC) criteria. Discrepancies were resolved through consensus, with unresolved disagreements adjudicated by the lead author (E. A. M.) to guide and validate final study selection.

### Organization and Data Extraction

A standardized data extraction tool was developed, piloted, and modified by the two primary reviewers in Google Sheets. The data extraction was conducted by one author and then validated by the other author. The information extracted included: title, authors, year of publication, country/location, methodological elements (study design, theoretical framework, population, sample size, and intervention if applicable), type of adversity reported, resilience dimension (process, capacity, or outcome), summary of findings, resilience facilitators, and resilience barriers. Resilience dimensions were categorized based on the definitions outlined in the PCC framework described above (See Supplementary File 1: Data Extraction Table). Any discrepancies during validation were resolved through consensus or by consulting the lead author. Consistent with earlier scoping review (Marfo et al., 2022) and recommended scoping review methodology (Peters et al., 2020), no formal quality appraisal of included studies was conducted, as the objective of this review was to map existing evidence rather than assess study quality.

### Summarizing and Reporting

Descriptive statistics using direct counts were conducted to report the frequencies and percentages of the characteristics of studies (e.g., year of publication, province, study design). We used a narrative description approach to synthesize the findings. Extracted data were mapped and grouped into overarching themes, paying close attention to intersectional factors (e.g., how the interaction of gender and immigrant status shaped a barrier). Age variability across studies was documented during data charting and handled descriptively during synthesis. Studies were grouped according to reported age ranges and population framing (adolescents only, young people aged 14–30 years, and mixed adult populations with maximum ages exceeding 30 years). Findings were synthesized narratively, with attention to population framing, and no direct age-based comparisons were conducted. The review was reported in accordance with the PRISMA Extension for Scoping Reviews (PRISMA-ScR) guidelines, and the completed checklist (Tricco et al., 2018) is provided in Supplementary File 2.

## Results

A total of 5,944 reports were initially retrieved from the database search using the original search strategy intended for the larger protocol. Following Level 1 and Level 2 screening against the PCC criteria, 11 articles met the inclusion criteria. An additional supplementary search on Google Scholar to capture Canada-context specific evidence and allowing flexibility in the age limit for studies focused primarily on young people yielded 12 additional studies that met the eligibility criteria. One student's thesis was identified from ProQuest Dissertations and Theses. Collectively, a total of 24 studies were included in this review, as summarized in the Preferred Reporting Items for Systematic Reviews and Meta-analysis for Scoping Reviews (PRISMA-ScR) flow chart (see Figure 1).

**Figure 1.**
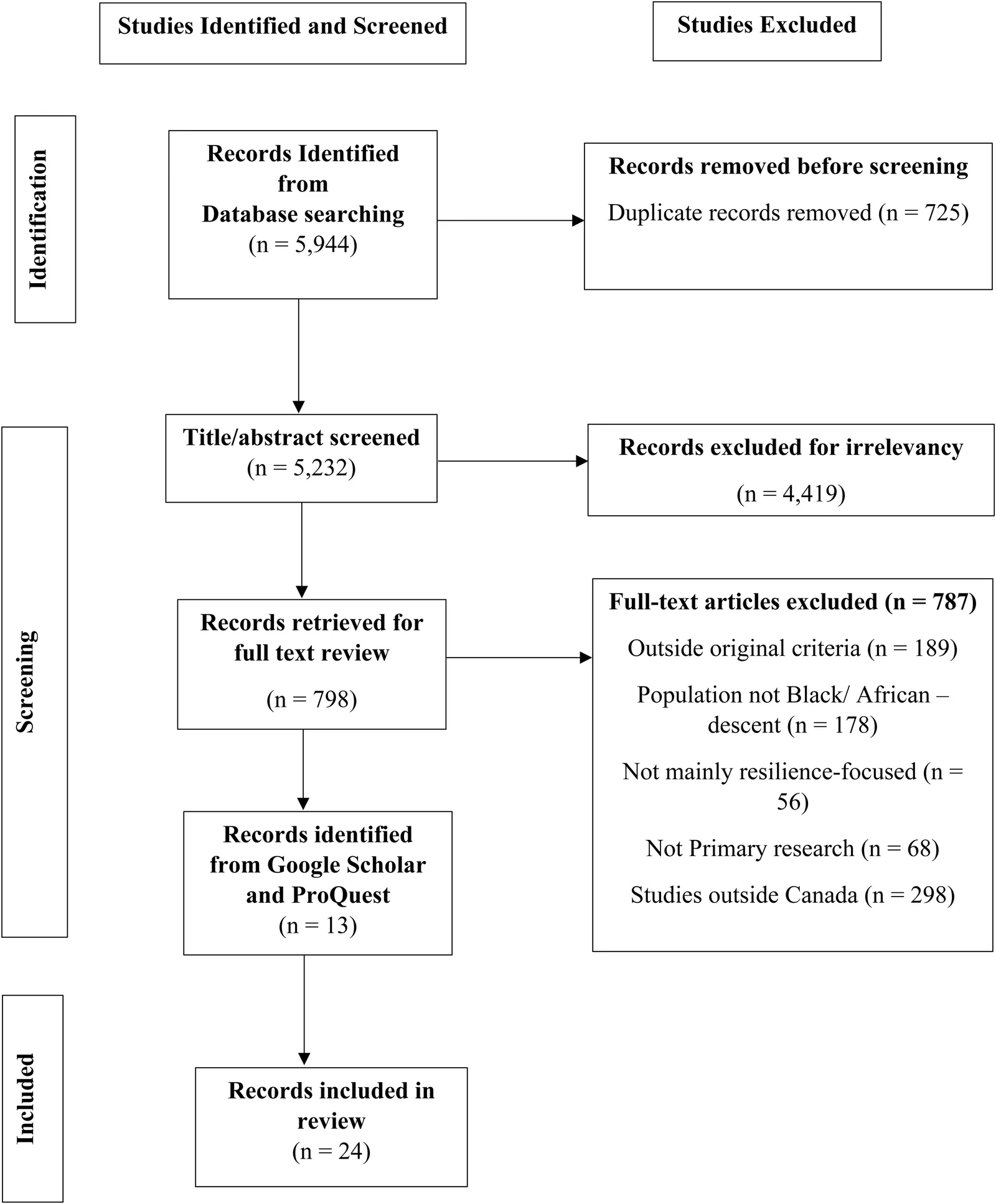
Flow diagram of study selection process.

### Study Characteristics

Most studies included in this review employed qualitative methodologies (18/24, 75%) while a smaller proportion used quantitative approaches (5/24, 20.8%). Sample sizes ranged from 4 to 1472 participants. The included studies were published between 2003 and 2025, with the majority (13/24, 54.2%) appearing from 2021 onwards. More than half of the studies were conducted in Ontario (15/24, 62.5%), followed by multi-provincial or national samples (6/24, 25%), with a few focusing on specific regions such as Eastern Canada or Saskatchewan (3/24, 12.5%). Regarding the population, the largest single category of focus was young people (14–30 years) (13/24, 54.2%). Most studies included participants both Canadian-born and first-generation immigrants (13/24, 54%), with fewer focusing exclusively on Canadian-born youth (5/24, 20.8%) or first-generation immigrants (3/24, 12.5%) (See Table 1).

**Table 1.** Summary of Characteristics of Included Studies (*N* = 24).

Characteristic	n (%)
*Year of Publication*	
2003-2010	3 (12.5)
2011–2020	4 (16.7)
2021–2025	17 (70.8)
*Territory / Province*	
Ontario only	15 (62.5)
Multi-provincial / national	6 (25.0)
Other provinces/regions	3 (12.5)
*Population group*	
Adolescents only (max age ≤18–19)	3 (12.5)
Young people (14–30 years)	13 (54.2)
Mixed adults (Max age > 30)	7 (29.2)
Not reported	1 (4.2)
*Sample size*	
<50	13 (54.2)
50–200	5 (20.8)
>200	6 (25.0)
*Methodological Approach*	
Qualitative	18 (75.0)
Quantitative	5 (20.8)
Mixed Methods	1 (4.2)
*Immigration Status*	
Canadian-born only	5 (20.8)
Immigrants	3 (12.5)
Mixed (Canadian-born and immigrants)	13 (54.2)
Not reported	3 (12.5)

### Theoretical Approaches in Included Studies

Studies included in this review demonstrate a strong emphasis on critical, trauma-informed, and resilience-based frameworks, often integrated to capture the complex, intersecting influences of race, oppression, and psychosocial factors on health and lived experiences. Critical Race Theory (CRT) and CRT-informed frameworks were most frequently applied across studies (Bollers & Eizadirad, 2025; George, 2023; Luhanga et al., 2023) and implied (Cénat et al., 2021). CRT was consistently used to examine systemic racism, anti-Black racism, and structural inequities, often combined with intersectionality, storytelling, feminist theory, and social determinants of health frameworks to explore the multi-dimensional effects of oppression (Edwards, 2025a; Yosieph, 2021).

Trauma-informed frameworks, including racial trauma and complex racial trauma theories, were used to examine psychological, emotional, and identity-related consequences of racialized adversity (Bailey et al., 2024; Cénat et al., 2025). Resilience-oriented frameworks emphasized coping processes, protective factors, and adaptive strategies at individual, relational, and community levels. Less frequently, studies drew on deliberative democracy, postcolonial theory, racelessness, and relational frameworks to explore civic engagement, identity negotiation, and empowerment. A small number of studies did not explicitly report a guiding theoretical framework.

### Contextual Adversities Framing Resilience and Resilience Dimensions Examined Across Studies

Across the included studies, resilience among Black children and young people was conceptualized in response to interconnected structural, systemic, interpersonal, and individual adversities. Structural and systemic barriers were most frequently highlighted, including anti-Black racism in education (Bailey et al., 2024; George, 2023), employment (Briggs, 2019; Hasford, 2016), healthcare (Edwards, 2025a; Laylor et al., 2025), and policing systems (Bailey et al., 2024; Osman et al., 2025). Socioeconomic precarity, housing instability, and exposure to community violence further compounded these challenges (Aiello, 2025; Logie et al., 2018) (see Supplementary File 3: Frequency of Contextual Adversities Framing Resilience Across Studies).

Despite these adversities, resilience was primarily operationalized as individual, relational, and community capacities that buffered against structural and interpersonal stressors. Commonly identified capacities included social support from family, peers, and mentors (Briggs, 2019; Gintova et al., 2025; Wilson-Forsberg et al., 2020), cultural identity and racial pride (Smith & Lalonde, 2003), emotional regulation and problem-solving skills (Cénat et al., 2021; Oluwasina et al., 2024), and participation in faith-based or community programs (Navara & Lollis, 2009).

Over half of the studies (18/24, 75%) conceptualized resilience as a dynamic process, highlighting adaptive strategies such as coping, resistance, meaning-making, and strategic identity negotiation in response to ongoing adversity (Aiello, 2025; Bollers & Eizadirad, 2025; Cénat et al., 2021; Luhanga et al., 2023). A smaller subset (3/24, 12.5%) measured resilience outcomes directly, using indicators such as psychological well-being, academic engagement, and identity safety (Cénat et al., 2021; Olawo et al., 2021; Smith & Lalonde, 2003).

Collectively, these findings illustrate that resilience among Black children and youth emerges in context: structural and interpersonal adversities shape both the challenges youth face and the capacities they draw upon to thrive. The interplay between adversities and resilience underscores the need for multi-level supports, including culturally responsive programs, mentorship, and safe spaces, to bolster adaptive functioning [see Supplementary File 3: Resilience Dimensions of Included Studies].

### Resilience Facilitators

Although Black children and young people face pervasive structural and systemic adversities, resilience was supported through interconnected individual, relational, and community-level protective factors that buffered these effects. At the community and relational level, social support was consistently identified as a key facilitator. Matrifocal networks and intergenerational storytelling preserved cultural continuity and identity (Navara & Lollis, 2009), while family support, peer networks, and mentorship from Black adults enhanced coping and adaptive functioning (Gintova et al., 2025; McCready & Junaid, 2025; Osman et al., 2025; Wilson-Forsberg et al., 2020). Religious participation and culturally relevant faith-based practices were important for fostering connectedness, perseverance, and self-worth (Edwards, 2025b; Navara & Lollis, 2009; Yosieph, 2021). Community-based programs, youth advisory involvement, safe spaces, and collective solidarity reinforced coping, identity safety, and empowerment (Bollers & Eizadirad, 2025; Laylor et al., 2025; McCready & Junaid, 2025).

At the individual level, internal capacities such as resilient coping, self-esteem, determination, problem-solving, life skills, and emotional regulation were critical facilitators. These capacities enabled youth to navigate racialized educational and social environments, manage psychosomatic symptoms, and maintain psychological well-being (Cénat et al., 2021; Logie et al., 2018; Osman et al., 2025). Adaptive strategies such as code-switching, strategic identity performance, reframing beliefs, and self-care supported youths’ ability to persist and resist discrimination (George, 2023; Hasford, 2016; Luhanga et al., 2023).

Cultural and identity-related factors also functioned as key facilitators. Strong cultural values, racial pride, cultural identity affirmation, and connection to heritage practices supported resilience by reinforcing a positive sense of self and belonging (Navara & Lollis, 2009; Smith & Lalonde, 2003; Wilson-Forsberg et al., 2020). Engagement in creative activities, advocacy, and exposure to diverse role models allowed Black youth to resist stereotypes and develop agency (Briggs, 2019; Edwards, 2025a; Roknuzzaman et al., 2024).

Finally, structural or programmatic supports emerged as facilitators in several studies. Access to culturally responsive programs, trauma-informed care, educational opportunities, supportive workplaces, and inclusion in decision-making fostered youth agency and provided environments in which resilience could be expressed and strengthened (Bollers & Eizadirad, 2025; Cénat et al., 2021; Edwards, 2025a; Gintova et al., 2025; Laylor et al., 2025; Oluwasina et al., 2024).

### Barriers to Resilience

Resilience among Black children and youth was constrained by a range of systemic, structural, interpersonal, and individual barriers that undermined protective mechanisms and limited adaptive responses to racialized adversity. Across multiple studies, experiences of racial discrimination and systemic racism were reported as pervasive obstacles.

In educational contexts, low teacher expectations, stereotyping, tracking into lower-level courses, and lack of representation in leadership positions eroded resilience and limited opportunities for achievement (Dromer et al., 2025; Gintova et al., 2025; Osman et al., 2025). Broader structural inequities such as precarious employment, economic vulnerability, food insecurity, and housing instability further constrained youths’ capacity to engage in protective behaviors and maintain psychological well-being (McCready & Junaid, 2025; Oluwasina et al., 2024).

Interpersonal dynamics also presented barriers to resilience. Intergenerational conflicts over values, gendered role expectations, and limited parental knowledge or involvement sometimes weakened family cohesion and youth agency (Navara & Lollis, 2009; Olawo et al., 2021). Peer exclusion, stereotyping, and microaggressions in school or community settings diminished youths’ sense of belonging and identity safety (George, 2023; Hasford, 2016; Luhanga et al., 2023). The absence of culturally affirming role models and mentorship restricted access to guidance and positive social capital (Bailey et al., 2024; Cénat et al., 2021).

At the individual level, internalized racism, performance anxiety, and bicultural identity stress undermined resilience, particularly when youth felt alienated from both their heritage and host cultures (Edwards, 2025b; Olawo et al., 2021; Smith & Lalonde, 2003). Stigma related to mental health, especially within faith-based or community settings, discouraged help-seeking behaviors (Edwards, 2025b; Yosieph, 2021). Experiences of trauma compounded psychological distress and limited adaptive coping strategies (Aiello, 2025; Osman et al., 2025).

Barriers were also apparent within programmatic and institutional contexts. Limited access to culturally relevant services, inconsistent program delivery, and systemic neglect of cultural identity in institutional settings created additional challenges for youth (Aiello, 2025; Bollers & Eizadirad, 2025; Laylor et al., 2025). Inconsistent support from social workers or educational staff, coupled with tokenistic or performative allyship, further hindered youth engagement and opportunities to build resilience (Edwards, 2025a; Osman et al., 2025).

### Identified Gaps from the Literature

Across the included studies, several gaps in the literature were identified. Limitations frequently included non-representative or small samples that constrained generalizability (Aiello, 2025; Cénat et al., 2025; Oluwasina et al., 2024). Several studies highlighted the need for clear definitions of racial discrimination and more precise conceptualizations of resilience to improve comparability across research (Cénat et al., 2025; Navara & Lollis, 2009).

There was a call for research incorporating diverse perspectives, including parental viewpoints, gendered socialization strategies, refugee experiences, and intergenerational or cross-national comparisons (Bailey et al., 2024; Wilson-Forsberg et al., 2020). Several studies noted a lack of longitudinal designs to understand causal relationships and the long-term impact of structural and interpersonal adversity on resilience (Aiello, 2025; George, 2023; Hasford, 2016; Oluwasina et al., 2024).

Other gaps pertained to insufficient exploration of institutional and structural factors, such as education, policing, and employment systems, and the absence of culturally responsive programs, safe spaces, and mentorship opportunities for Black children and youth (Cénat et al., 2021; Gintova et al., 2025; Laylor et al., 2025). Several studies also emphasized the need for research on mental health services that are culturally competent, accessible, and inclusive of spirituality and community contexts (Edwards, 2025b; Olawo et al., 2021; Smith & Lalonde, 2003).

## Discussion

This rapid scoping review aimed to synthesize Canadian literature to identify key facilitators and systemic barriers shaping resilience among Black children and young people. Our findings reveal: (a) geographic and methodological imbalances in research on resilience among this population; (b) that most studies contextualized resilience around intersecting structural and systemic adversities, including anti-Black racism in schools, socioeconomic hardship, and mental health barriers; (c) that research primarily explored resilience capacities and processes, with significantly less attention given to outcomes; (d) that resilience was supported by a dynamic combination of individual, relational, and community-level factors; and (e) that barriers to resilience spanned systemic, interpersonal, and individual levels, with racial discrimination and structural racism acting as pervasive obstacles. We discuss these findings in detail, followed by implications for future research, practice, and policy.

Most studies employed qualitative approaches, reflecting an emphasis on in-depth exploration of Black children and youth's lived experiences. These approaches generate rich, contextually grounded insights that capture the diversity of Black communities. However, the qualitative dominance, coupled with a paucity of longitudinal and interventional research, limits the ability to assess causal relationships or long-term impacts of structural adversities on resilience outcomes. This imbalance constrains policymakers’ capacity to justify systemic investments, which often rely on generalizable data.

A notable geographic skew was also observed, with over half of the studies conducted exclusively in Ontario. While Ontario is home to a large and diverse Black population (Statistics Canada, 2022), this concentration leaves a critical gap in our understanding of resilience in other Canadian provinces and territories. Differences in provincial education policies, policing practices, and mental health service availability may shape resilience differently across contexts. Future research must broaden its geographic scope to include the unique experiences of Black youth in the Prairies, Atlantic Canada, and particularly Francophone communities, to ensure that resilience frameworks are representative of the Canadian national context.

Also, in terms of theoretical approach, Critical frameworks predominated across the included studies, particularly Critical Race Theory (CRT) and Complex Racial Trauma frameworks, often integrated with intersectionality. These approaches reposition resilience not as an internal trait but as a response to anti-Black racism and structural inequities (Suslovic & Lett, 2024). The frequent integration of CRT with intersectionality suggests that researchers are increasingly recognizing that resilience is shaped by overlapping identities, such as gender and immigration status.

Resilience is rarely discussed in isolation; it is consistently framed within the context of structural and systemic adversities. Anti-Black racism in education, policing, healthcare, and social services forms the backdrop against which resilience emerges. By conceptualizing resilience as an active response to these adversities, rather than a deficit-correcting individual trait, studies challenge traditional deficit models that pathologize Black youth (Jones et al., 2023). Understanding resilience in this way emphasizes navigation and negotiation within hostile systems rather than simply measuring individual grit.

Resilience capacities and processes identified in the literature span individual, relational, and community domains. At the individual level, coping strategies, emotional regulation, problem-solving, and self-esteem were critical facilitators. Relationally, matrifocal networks, family support, mentorship, and peer networks reinforced adaptive functioning. Community and cultural factors, including intergenerational storytelling, faith-based practices, and collective solidarity, fostered identity safety, cultural pride, and perseverance (Boyd et al., 2022; Quattlebaum et al., 2025). These findings highlight that resilience is co-constructed through dynamic interactions across levels rather than residing solely within the individual.

Despite these protective factors, systemic, interpersonal, and individual barriers constrained resilience. Structural racism manifested as low teacher expectations, academic tracking, and limited access to culturally responsive services. Socioeconomic precarity and intergenerational conflicts further compounded adversity. Interpersonal microaggressions, lack of mentorship, and absence of culturally affirming role models restricted access to social capital. Individual-level challenges, such as internalized racism, bicultural identity stress, and mental health stigma, also limited adaptive capacities (Edwards, 2025b; Olawo et al., 2021). These findings illustrate that community support or individual “grit” alone is insufficient to overcome structural exclusion, highlighting the need for multi-level interventions.

A notable gap in the literature is the limited focus on resilience outcomes. While many studies examine capacities and processes, few assess long-term adaptation, such as academic success, mental health stability, or employment trajectories. This contrasts with earlier work (Wright et al., 2013), which emphasized observable outcomes. The divergence likely reflects differences in context and population, as our review included young people across adolescence and emerging adulthood in Canada. Bridging this gap requires research linking resilience processes with measurable outcomes over time.

## Implications for Research, Practice, and Policy

Future research should expand beyond Ontario-centric evidence base by prioritizing Black communities in Western Canada, the Maritimes, and Quebec, where distinct policy environments and service infrastructures may shape resilience for Black children and young people in different ways. Longitudinal and life-course approaches are urgently needed to examine how resilience capacities such as cultural pride, faith, and social support develop over time and translate into sustained adaptation outcomes, including educational attainment, employment trajectories, and long-term mental health. Furthermore, future research should adopt age-stratified designs to examine resilience mechanisms across developmental stages (e.g., early adolescence, late adolescence, emerging adulthood). Such approaches can illuminate how resilience evolves across age groups, inform developmentally tailored interventions, and clarify when and how protective factors exert their greatest influence. By prioritizing outcome-driven, age-sensitive research, scholars and policymakers can more effectively translate insights into actionable strategies that mitigate adversity and foster resilience.

Nursing and healthcare professionals must integrate cultural humility and relational inquiry into practice. Assessments should extend beyond the nuclear family to include matrifocal and intergenerational networks. Recognizing faith-based and spiritual practices as legitimate resilience resources enables holistic care that supports meaning-making and perseverance. Trauma-informed approaches should account for systemic mistrust, ensuring that clinical interactions do not inadvertently reproduce structural surveillance or marginalization.

Findings underscore the importance of structural reforms alongside community-level interventions. Policies should address systemic barriers in education, policing, and social services to prevent adversity from overwhelming protective capacities. Institutions must embed accountability mechanisms to ensure meaningful participation of Black youth in advisory and decision-making processes. Tokenistic allyship must be replaced with substantive, resourced initiatives that enhance resilience opportunities.

## Limitations and Strengths

Despite the methodological strengths, our rapid scoping review has some limitations. First, the search strategy was restricted to only English-language reports. Given Canada's bilingual status, this likely excluded significant evidence from Francophone Black communities, particularly in Quebec and New Brunswick. Second, while the initial database search (*n* = 11) followed the strict age parameters (0–24) of the larger cross-border protocol, we identified that this definition excluded a substantial body of Canadian literature regarding young Black adults. Consequently, our supplementary search yielded a higher volume of studies (*n* = 12) by expanding the age limit to studies that include young people. While this expansion was necessary to reflect the developmental and socioeconomic realities of Black youth in Canada, the divergence in search strategies between the primary and supplementary phases may limit the formal reproducibility of the search string. However, this adaptation was a deliberate methodological choice to ensure the review captured the full breadth of the Canadian-specific context. Furthermore, our inclusive age approach introduces conceptual heterogeneity across studies; therefore, findings should be interpreted as illustrative of resilience-related facilitators and barriers across broadly defined children and youth populations rather than as age-specific conclusions. Finally, due to the rapid nature of this scoping review, stakeholder consultation, as recommended by Levac et al. (2010), was not conducted. Thus, findings may require additional contextualization through future stakeholder engagement to guide actionable decision-making. However, our team included a Black young person whose perspectives meaningfully informed the data analysis.

A key strength of this review lies in its novel synthesis of multi-level facilitators and barriers to resilience among this population. Unlike prior work that often focuses mostly on mental health in general, this study highlights how systemic inequities intersect with personal and relational strengths to shape resilience. By linking community, relational, and structural factors in a single, comprehensive analysis, it provides a uniquely holistic perspective on resilience among Black youth in Canada. This integrative approach reinforces the importance of examining resilience as a dynamic process shaped by both context and individual agency, offering actionable insights for research, practice, and policy. Furthermore, moving beyond traditional developmental brackets (e.g., 15–24) to embrace the social and systemic definitions of “youth” allowed for a comprehensive synthesis of the adversities faced by Black young people during extended transitions into adulthood, particularly in the realms of employment and criminalization

## Conclusion

This review underscores that resilience among Black youth is deeply contextual, shaped by structural and systemic adversities rather than individual traits alone. Research remains predominantly descriptive, highlighting a critical gap in outcome-focused, age-stratified studies needed to inform interventions and policy. Addressing this gap will enable evidence-based strategies that not only support individual well-being but also challenge the systemic inequities that shape the lives of Black young people.

## Supplemental Material

sj-xlsx-1-cjn-10.1177_08445621261451565 - Supplemental material for A Rapid Scoping Review of Resilience Facilitators and Barriers among Black Children and Young People in Canada Supplemental material, sj-xlsx-1-cjn-10.1177_08445621261451565 for A Rapid Scoping Review of Resilience Facilitators and Barriers among Black Children and Young People in Canada by Emmanuel A. Marfo, Albert Junior Frimpong, Haris Musavar and Bukola Salami in Canadian Journal of Nursing Research

sj-docx-2-cjn-10.1177_08445621261451565 - Supplemental material for A Rapid Scoping Review of Resilience Facilitators and Barriers among Black Children and Young People in Canada Supplemental material, sj-docx-2-cjn-10.1177_08445621261451565 for A Rapid Scoping Review of Resilience Facilitators and Barriers among Black Children and Young People in Canada by Emmanuel A. Marfo, Albert Junior Frimpong, Haris Musavar and Bukola Salami in Canadian Journal of Nursing Research

sj-docx-3-cjn-10.1177_08445621261451565 - Supplemental material for A Rapid Scoping Review of Resilience Facilitators and Barriers among Black Children and Young People in Canada Supplemental material, sj-docx-3-cjn-10.1177_08445621261451565 for A Rapid Scoping Review of Resilience Facilitators and Barriers among Black Children and Young People in Canada by Emmanuel A. Marfo, Albert Junior Frimpong, Haris Musavar and Bukola Salami in Canadian Journal of Nursing Research

sj-docx-4-cjn-10.1177_08445621261451565 - Supplemental material for A Rapid Scoping Review of Resilience Facilitators and Barriers among Black Children and Young People in Canada Supplemental material, sj-docx-4-cjn-10.1177_08445621261451565 for A Rapid Scoping Review of Resilience Facilitators and Barriers among Black Children and Young People in Canada by Emmanuel A. Marfo, Albert Junior Frimpong, Haris Musavar and Bukola Salami in Canadian Journal of Nursing Research

## Data Availability

The data extraction table supporting the findings of this study is available as supplementary material.
