# Supplementary material for: A Rapid Scoping Review of Resilience Facilitators and Barriers among Black Children and Young People in Canada
Source: Can J Nurs Res. 2026 May 14;58(4):540–50. doi: 10.1177/08445621261451565 (PMC13714187; doi:10.1177/08445621261451565)
Supplement: Supplementary file 2 [file sj-docx-3-cjn-10.1177_08445621261451565.docx]

**Supplementary File 3: Frequency of Contextual Adversities Framing Resilience Across Studies (n = 24)**

**Resilience Dimensions Examined Across Studies**
