# Supplementary material for: A Rapid Scoping Review of Resilience Facilitators and Barriers among Black Children and Young People in Canada
Source: Can J Nurs Res. 2026 May 14;58(4):540–50. doi: 10.1177/08445621261451565 (PMC13714187; doi:10.1177/08445621261451565)
Supplement: Supplementary file 3 [file sj-docx-4-cjn-10.1177_08445621261451565.docx]

**Supplementary File 4: Resilience Dimensions of Included Studies**

| **Citation** | **How Resilience was Operationalized** | **Resilience Dimension (s)** |
| --- | --- | --- |
| Smith et al. (2003) | Psychological well-being, identity, academic engagement | Outcomes |
| Navara et al. (2009) | Family cohesion, cultural continuity, social support, adolescent agency | Capacities; Processes |
| Hasford et al. (2016) | Resistance strategies and adaptive responses | Capacities; Processes |
| Logie et al. (2018) | Resilient coping (belief in overcoming challenges) | Capacities |
| Wilson-Forsberg et al. (2018) | Cultural pride, adaptive coping, resistance to stereotypes | Capacities; Processes |
| Briggs (2019) | Collective resistance, brotherhood, solidarity | Capacities; Processes |
| Olawo et al. (2021) | Identity safety as a resilient outcome of migration | Processes; Outcomes |
| Cénat et al. (2021) | Social support as protective factor | Capacities; Processes; Outcomes |
| Yosieph (2021) | Identity, cultural resilience, gendered resilience | Capacities; Processes |
| Cénat et al. (2022) | Trait resilience (Resilience Scale-14) | Capacities |
| George et al. (2023) | Adaptive behaviors (code-switching, perseverance) | Processes |
| Luhanga et al. (2023) | Coping mechanisms (perseverance, determination) | Capacities; Processes |
| Bailey et al. (2024) | Enduring and transforming hardship | Capacities; Processes |
| Oluwasina et al. (2024) | BRS scores (low vs. normal/high resilience) | Capacities |
| Osman et al. (2024) | Youth engagement, empowerment, advocacy | Capacities; Processes |
| Aiello et al. (2025) | Adapting to ongoing gun violence and trauma | Processes |
| Bollers and Eisadirad, (2025) | Trauma-informed, culturally responsive programming | Capacities; Processes |
| Cénat et al. (2025) | Community resilience measured using Transcultural Community Resilience Scale | Capacities |
| Dromer et al. (2025) | Social support buffering mental health symptoms | Capacities |
| Edwards et al. (2025) | Art-based identity reconstruction and resistance | Capacities; Processes |
| Fiona (2025) | Spiritual, communal, and faith-based resilience | Capacities; Processes |
| Gintova et al. (2025) | Social support as buffering mechanism | Capacities; Processes |
| McCready and Junaid (2025) | Community support, empowerment, self-efficacy | Capacities; Processes |
| Laylor et al. (2025) | Relational resilience, youth agency, trust | Capacities; Processes |

NB: Some studies examined more than one resilience dimension
